# Training Girls

**Published:** 1871-08

**Authors:** 


### TRAINING GIRLS

For household duties ought to be considered as necessary as instruction in reading, writing, and arithmetic, and quite as universal. We are in our houses more than half of our existence, and it is the household surroundings which affect most largely the happiness or the misery of domestic life. If the wife knows how to "keep house," if she understands how to "set a table," if she has learned how things ought to be cooked, how beds should be made, how carpets should be swept, how the furniture should be dusted, how the clothing should be repaired, and turned, and altered, and renovated; if she knows how purchases can be made to the best advantage, and understands the laying in of provisions, how to make them go farthest and last longest; if she appreciates the importance of system, order, tidiness, and the quiet management of children and servants, then she knows how to make a little heaven of home, — how to win her children from the street; how to keep her husband from the club-house, the gaming-table, and the wine-cup; such a family will be trained to social respectability, to business success, and to efficiency and usefulness in whatever positions may be allotted to them.

It may be safe to say, that not one girl in ten in our large towns and cities enters married life, who has learned to bake a loaf of bread, to purchase a roast, to dust a painting, to sweep a carpet, or to cut and fit and make her own dress; how much the perfect knowledge of these things bears upon the thrift, the comfort, and the health of families, may be conjectured, but not calculated by figures. It would be an immeasurable advantage to make a beginning by attaching a kitchen to every girls' school in the nation, and have lessons given daily in the preparation of all the ordinary articles of food and drink for the table, and how to purchase them in the market to the best advantage, with the result of a large saving of money, an increase of comfort, and higher health in every family in the land.

